# Dielectrophoretic Microfluidic Device for Separating Microparticles Based on Size with Sub-Micron Resolution

**DOI:** 10.3390/mi11070653

**Published:** 2020-06-30

**Authors:** Salini Krishna, Fadi Alnaimat, Ali Hilal-Alnaqbi, Saud Khashan, Bobby Mathew

**Affiliations:** 1 Mechanical Engineering Department, United Arab Emirates University, Al Ain P.O. Box 15551, UAE; 201990211@uaeu.ac.ae (S.K.); falnaimat@uaeu.ac.ae (F.A.); 2 Abu Dhabi Polytechnic, MBZ Campus, United Arab Emirates, Abu Dhabi P.O. Box 111499, UAE; ali.alnaqbi@adpoly.ac.ae; 3 Mechanical Engineering Department, Jordan University of Science and Technology, Irbid 22110, Jordan; sakhashan@just.edu.jo; 4 Zayed Center for Health Sciences, United Arab Emirates University, Al Ain P.O. Box 15551, UAE

**Keywords:** dielectrophoresis, microchannel, modeling, separation, separation efficiency, separation purity

## Abstract

This article details the mathematical model of a microfluidic device aimed at separating any binary heterogeneous sample of microparticles into two homogeneous samples based on size with sub-micron resolution. The device consists of two sections, where the upstream section is dedicated to focusing of microparticles, while the downstream section is dedicated to separation of the focused stream of microparticles into two samples based on size. Each section has multiple planar electrodes of finite size protruding into the microchannel from the top and bottom of each sidewall; each top electrode aligns with a bottom electrode and they form a pair leading to multiple pairs of electrodes on each side. The focusing section subjects all microparticles to repulsive dielectrophoretic force, from each set of the electrodes, to focus them next to one of the sidewalls. This separation section pushes the big microparticles toward the interior, away from the wall, of the microchannel using repulsive dielectrophoretic force, while the small microparticles move unaffected to achieve the desired degree of separation. The operating frequency of the set of electrodes in the separation section is maintained equal to the cross-over frequency of the small microparticles. The working of the device is demonstrated by separating a heterogeneous mixture consisting of polystyrene microparticles of different size (radii of 2 and 2.25 μm) into two homogeneous samples. The mathematical model is used for parametric study, and the performance is quantified in terms of separation efficiency and separation purity; the parameters considered include applied electric voltages, electrode dimensions, outlet widths, number of electrodes, and volumetric flowrate. The separation efficiencies and separation purities for both microparticles are 100% for low volumetric flow rates, a large number of electrode pairs, large electrode dimensions, and high differences between voltages in both sections.

## 1. Introduction

Microfluidic devices are those devices with flow passages smaller than 1000 μm, and this brings about certain advantages including a reduced need for sample and reagents, reduced power consumption, portability, and small footprint [1,2]. Additionally, microfluidic devices allow for enabling phenomena that are often not practically realizable in any device of conventional length scales [3]. One of the applications for which microfluidic devices are employed includes the separation of a heterogeneous mixture of microparticles into multiple homogeneous samples; the homogeneity could be in terms of size or type. In order to achieve separation, every microparticle in the heterogeneous sample needs to be acted upon by an actuation force and, preferably, it should be non-invasive. Several phenomena are currently employed in microfluidic devices for generating the desired non-invasive actuation force [4]. Dielectrophoresis (DEP) is one phenomenon that is employed in microfluidic devices for purposes of separation of samples [4,5,6,7]. DEP is ideally suited as an actuation phenomenon in microfluidic devices as it scales well with miniaturization and can be realized without requiring specialized wafers. DEP is the phenomenon that describes the movement of microparticles when exposed to a spatially varying electric field while being suspended in a dielectric medium [4,5,6,7]. The movement is toward either the maxima or the minima of the gradient of the electric field, and the force associated with DEP is presented in Equation (1) [4,5,6,7]. The movement of a microparticle toward the maxima of the gradient of the electric field is specifically termed as positive-DEP or pDEP, while the movement of a microparticle toward the minima of the gradient of the electric field is specifically termed as negative-DEP or nDEP. The preference of a microparticle for the maxima or minima is influenced by the properties (conductivity and permittivity) of the medium and microparticle, as well as the operating frequency of the electric signal. The combined effect of the properties (of the microparticle and medium) and the operating frequency is included in the Clausius–Mossotti factor, Re[*f*_CM_], which is mathematically stated in Equation (2); the electrical conductivity of microparticles is dependent on the bulk conductivity and surface conductance as shown in Equation (3) [8]. For Re[*f*_CM_] > 0 and Re[*f*_CM_] < 0, the microparticle will experience pDEP and nDEP, respectively; for Re[*f*_CM_] = 0, the microparticle will not experience DEP. For a particular combination of microparticle (fixed properties) and medium (fixed properties), the polarity of Re[*f*_CM_] can be varied by changing the operating frequency; the operating frequency for which a microparticle does not experience DEP is given in Equation (4), and this frequency is referred to as cross-over frequency (*N_cr_*).

(1) $$F_{DEP}=2\pi \varepsilon_{m}r_{e}^{3}Re\left[f_{CM}\right]\nabla E_{RMS}^{2}.$$

(2) $$Re\left[f_{CM}\right]=\frac{4\pi^{2}N^{2}\left(\varepsilon_{e}+2\varepsilon_{m}\right)\left(\varepsilon_{e}-\varepsilon_{m}\right)+\left(\sigma_{e}+2\sigma_{m}\right)\left(\sigma_{e}-\sigma_{m}\right)}{4\pi^{2}N^{2}{\left(\varepsilon_{e}+2\varepsilon_{m}\right)}^{2}+{\left(\sigma_{e}+2\sigma_{m}\right)}^{2}}.$$

(3) $$\sigma_{e}=\sigma_{bulk}+2\frac{K_{s}}{r_{e}}.$$

(4) $$N_{cr}=\frac{1}{2\pi }\sqrt{\frac{\left(\sigma_{e}+2\sigma_{m}\right)\left(\sigma_{m}-\sigma_{e}\right)}{\left(\varepsilon_{e}+2\varepsilon_{m}\right)\left(\varepsilon_{e}-\varepsilon_{m}\right)}}.$$

**Figure 1:**
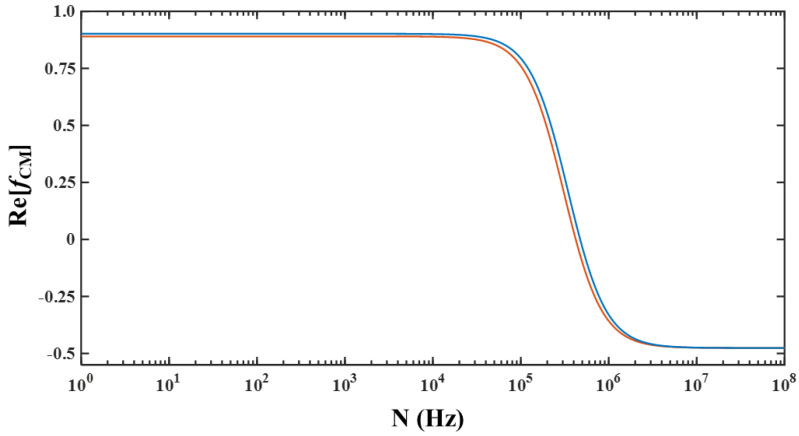
Variation of *Re*[*f*_CM_] with operating frequency (*N*) for 2-μm (■) and 2.25-μm (■) microparticles.

Figure 1 shows the variation of Re[*f*_CM_] with operating frequency for polystyrene microparticles (*ε_e_* = 2.55ε_o_, *K_s_* = 2.85 nS, *ε_o_* = 8.8452 pF/m) with a radius of 2 μm and 2.25 μm suspended in water (*ε_m_* = 78.5*ε_o_*, *σ_m_* = 10^−4^ S/m) [8,9]. It can be noticed that both microparticles exhibit pDEP and nDEP at low and high frequencies, respectively. Moreover, it can be noticed that the cross-over frequency of the 2-μm microparticles is higher than the cross-over frequency of the 2.25-μm microparticles. Based on Equation (4), the cross-over frequencies of 2-μm and 2.25-μm microparticles are ca. 473 kHz and ca. 421 kHz, respectively.

**Figure 2:**
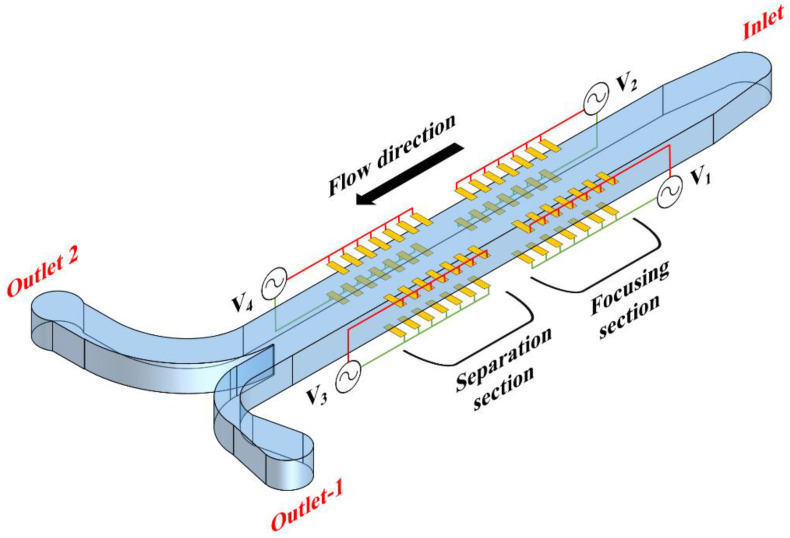
Schematic of the proposed microfluidic device (perspective view).

The device proposed in this document for purposes of separating a binary heterogeneous sample of microparticles into two homogeneous samples is shown in Figure 2. The device consists of one inlet and two outlets; the flow in the device is unequally split between the outlets. It can be noticed that the device consists of an upstream section, wherein microparticles are focused, and a downstream section, wherein the focused microparticles are separated into two samples. The focusing section consists of multiple finite-sized electrodes protruding into the microchannel from the top and bottom of both the sidewalls. The electrodes on the top of each sidewall are aligned with the electrodes on the bottom of the same sidewall. Every electrode protruding into the microchannel from the top and bottom of the same sidewall forms a pair; thus, there are multiple electrode pairs on both sides of the microchannel, as shown in Figure 2. The applied electrical potential is kept the same for all the electrode pairs of a particular side of the microchannel. With regard to Figure 2, the applied electrical potentials are *V*_1_ and *V*_2_. The separation section has a similar arrangement of electrodes as the focusing section (Figure 2); the applied electric potentials are *V*_3_ and *V*_4_.

**Figure 3:**
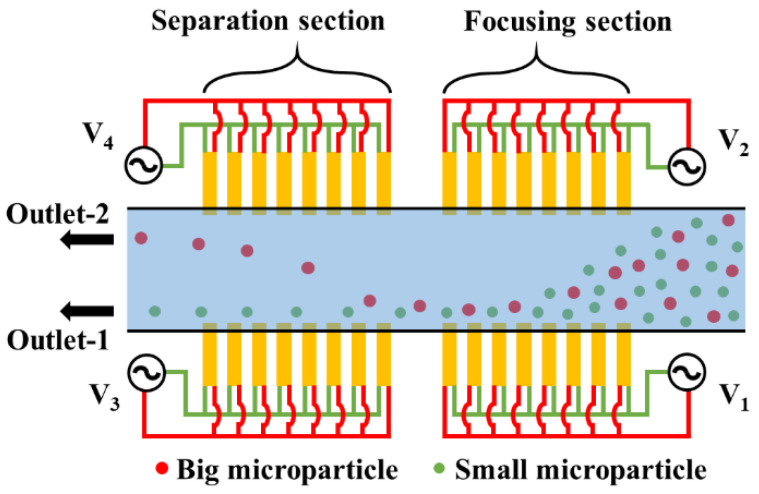
Schematic of the working of the device (top view); in the focusing section, the heterogeneous mixture is subjected to negative dielectrophoresis (nDEP) forces from both the electrode pairs, leading to their focusing near one set of the electrode pairs, whereas, in the separation section, the big microparticles are subjected to nDEP forces leading to them being pushed into the interior of the microchannel while the small microparticles do not experience DEP, leaving their position unaffected; *V*_1_ < *V*_2_, *V*_3_ > *V*_4_, *N*_1_ = *N*_2_ >> *N_cr_*, *N*_3_ = *N*_4_ = *N_cr,sp._*

Figure 3 shows the working of the microfluidic device conceptualized in Figure 2. The applied electric potentials (*V*_1_ and *V*_2_) associated with the focusing section are different; nevertheless, all electrode pairs subject microparticles to nDEP. The nDEP force associated with the higher applied electric potential (*V*_2_) is greater than the nDEP force associated with the lower applied electric potential (*V*_1_), and this allows focusing the microparticles next to one of the sidewalls. The microparticles are focused next to the electrode pairs with the lower applied electric potential. The operating frequencies associated with the focusing section are kept very high (>10 MHz) so that Re[*f*_CM_] is negative. In the separation section, the applied electrical potentials are different with *V*_3_ being higher than *V*_4_. Moreover, the operating frequencies in the separation section are kept equal to the cross-over frequency of the small microparticles so that they do not experience DEP, while the other microparticles experience nDEP. The nDEP force experienced by the big microparticles is greater from the electrode pairs with the applied electric potential of *V*_3_ compared with the nDEP force experienced by the big microparticles from the electrode pairs with the applied electric potential of *V*_4_. The net nDEP force experienced by the big microparticles will, thus, push it toward the interior, away from wall, of the microchannel while the small microparticles move through separation section unaffected, thereby achieving the desired degree of separation. This pushing of the big microparticles into the interior, away from the wall, of the microchannel will lead them to be positioned in streamlines that progress toward outlet-2. At the same time, the small microparticles remain positioned in streamlines that progress toward outlet-1. It is stressed here that there will be no mixing of microparticles beyond the separation section as the device operates in the laminar flow regime.

This document is the first to propose the device shown in Figure 2. The proposed device is easy to fabricate, compared with devices with vertical or liquid electrodes, as the electrodes are planar [10,11]. Additionally, the world-to-chip electrical connection for the proposed electrode configuration is less complex than that required for interdigitated transducer (IDT) electrodes, and this allows for having a high number of electrode pairs in the device. The proposed microfluidic device can handle high throughputs as well, because the microparticles can be subjected to DEP over a great distance.

Kralj et al. [12] modeled and constructed a microfluidic device for the separation of microparticles based on size. The device employed slanted planar IDT electrodes. The microfluidic device has three inlets with one inlet used for introducing the binary heterogeneous mixture while the other two inlets introduce sheath flow. The microparticles are focused near one of the sidewalls using the sheath flows prior to being acted upon by nDEP force. As the nDEP force depends on the size of the microparticles, the bigger microparticles are pushed further into the interior of the microchannel than smaller microparticles, thereby achieving the required separation based on size. Kralj et al. [12] developed an experimentally validated model for this device, and it included the effect of drag and DEP but neglected inertia. Han and Frazier [11] developed two microfluidic devices with V-shaped planar electrodes, arranged in interdigitated transducer configuration, on the bottom surface of the microchannel for type-based separation of cells. Separation is achieved in this device by subjecting all cells to nDEP with one type of cells experiencing greater nDEP force compared with the other type. Han and Frazier [11] demonstrated the efficiency of the devices by separating a heterogeneous mixture of red blood cells (RBCs) and white blood cells (WBCs) into homogeneous samples of RBCs and WBCs. Wang et al. [13] developed a microfluidic device with two sets of vertical electrodes in IDT configuration for achieving separation based on type; each set of electrodes is located on one of the sidewalls. Each set of electrodes is operated at a unique applied electric potential and operating frequency. Thus, the net DEP force experienced by microparticles, in the microfluidic device, is type-dependent, thereby allowing for achieving separation based on type. Wang et al. [13] developed a static model of the microfluidic which allows for determining the equilibrium position of microparticles, and it is dependent on the Re[*f*_CM_] and applied electrical potential of both sets of electrodes. Lewpiriyawong et al. [14] constructed a microfluidic device that employed sheath flow and DEP for separation of microparticles based on size. Sheath flow focused the heterogeneous mixture of microparticles, prior to being subjected to DEP, next to one of the sidewalls. Several vertical electrodes placed on this sidewall, in IDT configuration, subject the microparticles to nDEP, which pushes them into the interior of the microchannel. The big microparticles are pushed further into the microchannel than small microparticles, and this leads to the separation of the heterogeneous mixture of microparticles. Lewpiriyawong et al. [14] developed a two-dimensional (2D) model of the microfluidic device which included the influence of several phenomena including inertia, drag, and DEP; a 2D as opposed to a three-dimensional (3D) model was used as there is no variation of electrical parameters along the depth of the microchannel. Altinagac et al. [15] developed a microfluidic device with slanted IDT planar electrodes for the purpose of size-based separation of microparticles. In the device, the operating frequency of the alternating current is selected such that the big microparticles experience nDEP while the small microparticles do not experience DEP. Thus, the small microparticle passes over the electrodes unaffected while the big microparticle is pushed along the width, of the microchannel, by nDEP force, thereby achieving separation based on size. Alazzam et al. [16] modeled the working of a microfluidic device with multiple finite-sized electrodes placed on the top and bottom surfaces of the microchannel; the electrodes on the top surface align with the electrode gaps on the bottom surface. All microparticles are subjected to nDEP causing their levitation; the levitation height is a function of the permittivity and density of the microparticle and medium, and this allows for separation of microparticles based on type. The model accounted for several phenomena such as inertia, drag, gravity, buoyancy, and DEP. The model was used for parametric study. Ali and Park [17] modeled a microfluidic device with liquid electrodes for type-based separation of a heterogeneous mixture of white blood cells (WBCs), red blood cells (RBCS), and platelets. The device consists of multiple liquid electrodes placed next to one of the sidewalls. The incoming stream of cells are focused, close to the sidewall next to the liquid electrodes, by sheath flow and subsequently subjected to nDEP. The nDEP force caused lateral displacement of the entities which varied depending on the type, thereby achieving separation based on the same. The model accounted for the influence of phenomena such as inertia, drag, gravity, buoyancy, and DEP. Ali and Park [17] studied the influence of several operating and geometric parameters, using the model on the performance of the device. Alnaimat et al. [8] modeled the functioning of a microfluidic device, with planar IDT electrodes on the bottom surface of the microchannel, employed for type-based separation. This frequency of operation is selected such that one type of microparticle is subjected to pDEP while the other type of microparticle is subjected to nDEP. The microparticles subjected to pDEP are attracted and captured on the electrodes, while the microparticles experiencing nDEP are levitated inside the microchannel, thereby achieving the desired separation. The model took into consideration the influence of phenomena such as inertia, drag, gravity, buoyancy, and DEP. The model was used for parametric study. Tajik et al. [18] developed a microfluidic device with four right-triangle shaped electrodes; two electrodes are placed on the top surface while the other two electrodes are placed on the bottom surface of the microchannel. Each electrode is positioned with one edge in contact with one of the sidewalls and a second edge perpendicular to the same sidewall; additionally, the leading-edge width of the electrode is zero. Each top electrode is aligned with the bottom electrode on the same side of the microchannel. With this electrode configuration, type-based separation is achieved by subjecting one type of microparticle to pDEP, which is subsequently drawn to the region between the top and bottom electrodes, while the other type of microparticle is acted upon by nDEP to be pushed toward the center of the microchannel. Tajik et al. [18] modeled the microfluidic device by including the influence of phenomena such as drag and DEP.

This work presents the first attempt at modeling the microfluidic device, shown in Figure 2, working under the proposed scheme. The mathematical model takes into account several forces such as those associated with inertia, gravity, buoyancy, drag, virtual mass, and DEP. The inclusion of forces associated with inertia and drag makes the model dynamic, thereby allowing the quantification of the temporal variation of the trajectory of the microparticles. Additionally, the dynamic nature of the model allows for determining the length, as well as the number of electrode pairs required for creating a device with the desired level of performance metrics; this would not be possible using a static model. The model developed for the proposed microfluidic device is three-dimensional, thereby allowing to account for microparticle’s displacement along the height of the microchannel; this is crucial when handling microparticles with density different from that of the medium and is, thus, a merit of the model.

## 2. Mathematical Modeling

The mathematical model of the microfluidic device is described in this section. The model consists of multiple equations as provided below. The fluid flow through the microchannel is described by the continuity equation, Equation (5), and the Navier–Stokes equation, Equation (6) [19]. The electric potential inside the microchannel is described using the Laplace equation, Equation (7), and the relationship between electric potential and electric field is provided below as well in Equation (8) [19]. The motion of the microparticle is described by Newton’s second law, shown in Equation (9), [19]. Joule heating is considered negligible for the electrical conductivity considered in this study and, thus, the energy equation is not included in the model [20].

(5) $$\nabla \cdot U_{m}=0.$$

(6) $$U_{m}\cdot \nabla U_{m}=-\frac{1}{\rho_{m}}\nabla P+\frac{\mu_{m}}{\rho_{m}}∆U_{m}.$$

(7) $$∆V_{RMS}=0.$$

(8) $$E_{RMS}=-\nabla V_{RMS}.$$

(9) $$m_{e}\frac{d}{dt^{2}}X_{e}=\sum^{}F_{e,ext}.$$

The fluid flow in the microchannel is fully developed, i.e., with finite velocity in the axial direction, while lateral velocities are non-existent, from the start of the electrodes of the focusing section. For fully developed flow, the Navier–Stokes equations are reduced to the equation shown below in Equation (10); the solution to Equation (10) is provided in Equation (11) [5].

(10) $$\frac{\partial^{2}u_{m}}{\partial {\bar{y}}^{2}}+\frac{\partial^{2}u_{m}}{\partial {\bar{z}}^{2}}=\frac{1}{\mu_{m}}\frac{dP}{d\bar{x}}.$$

(11) $$u_{m}\left(x_{e},y_{e},z_{e}\right)=\frac{48Q_{m}}{\pi^{3}W_{ch}H_{ch}}\frac{\sum_{i=1,3,5}^{\infty }\left(\frac{{\left(-1\right)}^{\left(\frac{i-1}{2}\right)}}{i^{3}}\right)cos\left[i\frac{\pi }{W_{ch}}\left(\frac{W_{ch}}{2}-y_{e}\right)\right]\left\{1-\frac{cosh\left[i\frac{\pi }{W_{ch}}\left(\frac{H_{ch}}{2}-z_{e}\right)\right]}{cosh\left(\frac{i\pi }{2}\frac{H_{ch}}{W_{ch}}\right)}\right\}}{\left[1-\frac{192\;W_{ch}}{\pi^{5}H_{ch}}\sum_{i=1,3,5}^{\infty }\frac{tanh\left(i\frac{\pi }{2}\frac{H_{ch}}{W_{ch}}\right)}{i^{5}}\right]}.$$

**Figure 4:**
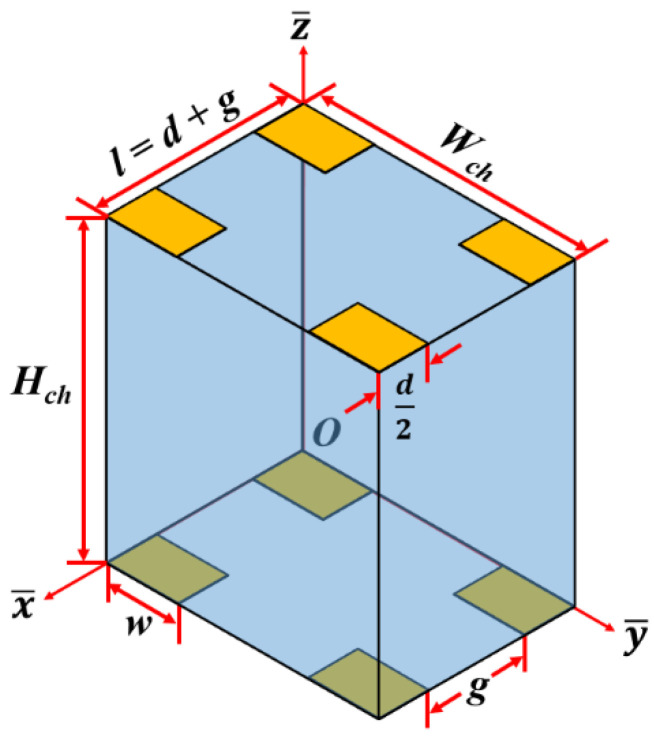
Schematic of the repeating unit for both focusing and separation sections (*H_ch_*: microchannel height, *W_ch_*: microchannel width, *l*: repeating unit length, *w*: electrode length, *d*: electrode width, *g*: gap between electrodes, and *O*: origin).

Equation (11) presents the axial velocity at any cross-section of the microchannel when the boundary conditions associated with Equation (10) include zero axial velocity on the walls of the microchannel. There is no analytical solution for Equation (7) when it is used for representing the electric potential inside the focusing and separation sections of the proposed microfluidic device; Equation (7) is solved using the finite difference method (FDM). As can be noticed from Figure 2, the focusing and separation sections consist of multiple pairs of electrodes. Nevertheless, each section can be considered to be made up of repeating units presented in Figure 4. Equations (7) and (8) are, thus, solved to obtain the electric potential and electric field in a single repeating unit, respectively; information about electric potential and electric field associated with a single repeating unit is subsequently mapped onto all the repeating units that make up the focusing and separation sections. A similar approach is adopted as well with regard to determining the DEP force. This approach neglects the end effects at the boundaries along the axial direction of the focusing and separation section; nevertheless, the end effects are negligible for a high number of electrode pairs as is in the case of the conceptualized device. The boundary conditions associated with Equation (7) include known voltages on the electrode surfaces and zero electric field on the remaining surfaces of the repeating unit. For implementing FDM, each repeating unit is initially populated with nodes; the internode distance is maintained at 1 μm in all directions. Afterward, Equation (7) is converted into a difference equation, by replacing the differential terms with second-order central difference terms, and it is applied to each node, leading to the generation of a system of linear equations which, upon solving, will provide the electric potential at the nodes [19]. The system of linear equations is solved using the Gauss–Seidel method [21]. Equation (8) is numerically evaluated as well; for this, the differential terms are replaced by difference terms and applied to each of the nodes to determine the electric field at the same location. Once the electric field at the nodes is determined, the DEP force at the same nodes is calculated by replacing the differential terms of Equation (1) by difference terms. The DEP force at any location other than the nodes is determined through interpolation using the DEP force of the nodes surrounding the location of interest. A second-order central difference scheme is used for replacing the differential terms of Equations (1) and (8) [19]. On the other hand, second-order backward/center/forward difference schemes are used for replacing the differential terms of Equations (1) and (8) [19].

The external forces acting on the microparticle include those associated with gravity, buoyancy, virtual mass, DEP, and drag. Forces associated with gravity and buoyancy act only in the vertical direction; however, the other forces act in all directions. Summation of all forces acting on the microparticle along the *x*-direction (Equation (12)), *y*-direction (Equation (13)), and *z*-direction (Equation (14)) are provided below. The first term on the right-hand side of Equations (12), (13), and (14) represent force associated with drag, while the second and third terms on the right-hand side of the same equations represent the force related to DEP and virtual mass, respectively. The fourth terms on the right-hand side of Equation (14) represent the sedimentation force, i.e., difference between forces associated with gravity and buoyancy. The relative important of these forces was analyzed by Castellanos et al. [22].

(12) $$\sum^{}F_{e,ext,\bar{x}}=6\pi \mu_{m}r_{e}\left({u_{m}|}_{X_{e}}-\frac{dx_{e}}{dt}\right)+2\pi \varepsilon_{m}r_{e}^{3}Re\left[f_{CM}\right]{\frac{\partial E_{RMS}^{2}}{\partial \bar{x}}|}_{X_{e}}-\frac{2}{3}\rho_{m}\pi r_{e}^{3}\left(\frac{d^{2}x_{e}}{dt^{2}}-{\frac{du_{m}}{dt}|}_{X_{e}}\right).$$

(13) $$\sum^{}F_{e,ext,\bar{y}}=-6\pi \mu_{m}r_{e}\frac{dy_{e}}{dt}+2\pi \varepsilon_{m}r_{e}^{3}Re\left[f_{CM}\right]{\frac{\partial E_{RMS}^{2}}{\partial \bar{y}}|}_{X_{e}}-\frac{2}{3}\rho_{m}\pi r_{e}^{3}\left(\frac{d^{2}y_{e}}{dt^{2}}-{\frac{du_{m}}{dt}|}_{X_{e}}\right).$$

(14) $$\sum^{}F_{e,ext,\bar{z}}=-6\pi \mu_{m}r_{e}\frac{dz_{e}}{dt}+2\pi \varepsilon_{m}r_{e}^{3}Re\left[f_{CM}\right]{\frac{\partial E_{RMS}^{2}}{\partial \bar{z}}|}_{X_{e}}-\frac{2}{3}\rho_{m}\pi r_{e}^{3}\left(\frac{d^{2}x_{e}}{dt^{2}}-{\frac{du_{m}}{dt}|}_{X_{e}}\right)-\frac{4}{3}\pi g_{a}r_{e}^{3}\left(\rho_{e}-\rho_{m}\right).$$

Equation (9) is solved using FDM as well. The differential terms are replaced by second-order central difference schemes, which allows for converting the differential equations into difference equations. The time step of the difference equations is maintained at 10^−5^ s. The boundary conditions associated with Equation (8) include the initial displacement and initial velocities [19].

The performance of the microfluidic device is quantified in terms of separation efficiency (SE) and separation purity (SP). SE is the ratio of the number of microparticles of a particular size reaching the designated outlet of the microfluidic device to the number of the microparticles of the same size introduced at the inlet of the microfluidic device. SP is the ratio of the number of microparticles of a particular size reaching the designated outlet of the microfluidic device to the total number of microparticles reaching the same outlet of the microfluidic device. Both SE and SP are mathematically stated in Equations (15) and (16), respectively. Several microparticles, uniformly distributed across the inlet of the microchannel, are released from the inlet, and the trajectory of each microparticle is tracked to calculate SE and SP; microparticles are released from 81 locations across the inlet of the microchannel.

(15) $$SE\left(A\right)=\frac{\#\;of\;microparticles\;of\;size-A\;at\;outlet\;designated\;for\;microparticle\;of\;size-A}{\#\;of\;microparticles\;of\;size-A\;at\;inlet}.$$

(16) $$SP\left(A\right)=\frac{\#\;of\;microparticles\;of\;size-A\;at\;outlet\;designated\;for\;microparticle\;of\;size-A}{\#\;of\;all\;microparticles\;at\;outlet\;designated\;for\;micropaticle\;of\;size-A}.$$

## 3. Results and Discussion

**Figure 5:**
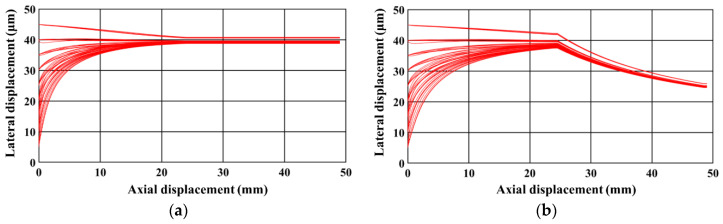
Top view of the trajectory of the (**a**) 2-μm microparticles and (**b**) 2.25-μm microparticles (*d_f_* = 60 μm, *l_f_* = 120 μm, *d_s_* = 60 μm, *l_s_* = 120 μm, *Q_m_* = 200 μl/h, *w_f_* = 6 μm, *w_s_* = 6 μm, *n_f_* = 200, *n_s_* = 200, *V*_1_ = 3 V_pp_, *V*_2_ = 15 V_pp_, *V*_3_ = 15 V_pp_ *V*_4_ = 3 V_pp_, *H_ch_* = 50 μm, *W_ch_* = 50 μm, 0 < *W*_*o*,2.25_ < 30 μm, 30 μm < *W*_*o*,2_ < 50 μm, *N*_1_ = *N*_2_ = 10 MHz, *N*_3_ = *N*_4_ = *N*_*cr*,2_ = 473 kHz).

The first part of this section demonstrates the ability of the microfluidic device in achieving separation based on size with sub-micron resolution; for this, the model is used for demonstrating the ability of the device in separation a heterogeneous mixture of 2-μm (radius) and 2.25-μm (radius) polystyrene (*ρ_e_* = 1050 kg/m^3^) microparticles suspended in water (*ρ_m_* (at 20 °C) = 998 kg/m^3^, *μ_m_* (at 20 °C) = 10^−3^ Pa∙s), based on size [8]. Figure 4 shows the trajectory (top view) of microparticles inside the microfluidic device. Figure 5a,b present the top view of the trajectory of 2-μm and 2.25-μm microparticles, respectively. It can be noticed that both 2-μm and 2.25-μm microparticles are similarly focused in the focusing section of the microfluidic device; the microparticles are focused close to one of the sidewalls. For this, the electrode pairs on both sides of the microfluidic device are operated at very high frequency (>10 MHz) and, thus, the Re[*f*_CM_] is −0.476. Moreover, the nDEP force is greater from the electrode pairs on one side of the microchannel compared with the nDEP force from the electrode pairs on the other side of the microchannel; this difference in nDEP is achieved by keep the applied electrical potentials unequal. The microparticles are focused next to the electrodes with the lower applied electrical potential. On the other hand, the operating frequencies of all electrode pairs, in the separation section, are maintained at or very close to the cross-over frequency of the 2-μm microparticles and, thus, they do not experience any DEP force. Nevertheless, the 2.25-μm microparticles experience nDEP force, causing them to move toward the interior of microchannel. Subsequently, the 2-μm microparticles exit the microfluidic device through an outlet to which all streamlines between the width of 30 μm and 50 μm progress, while the 2.25-μm microparticles exit the microfluidic device through another outlet to which all streamlines between the widths of 0 and 30 μm progress and, thus, the desired separation of the heterogeneous sample is achieved. The separation and focusing sections are separated by 500 μm; there are no electrodes in this region.

In this part of this section of the article, the influence of operating and geometric parameters on SE and SP of the microparticles is studied. The operating and geometric parameters considered include electrode dimensions (*w_f_*/*w_s_* and *d_f_*/*d_s_*), number of electrodes (*n_f_*/*n_s_*), volumetric flow rate (*Q_m_*), and applied electric potentials (*V*_*pp*1_/*V*_*pp*2_ and *V*_*pp*3_/*V*_*pp*4_). For parametric study, one of the parameters is varied while all other parameters are kept constant and, subsequently, the corresponding SE and SP are calculated. For all parametric studies, 2-μm and 2.25-μm microparticles suspended in water are employed; the operating frequency of the focusing section is 10 MHz while that of the separation section is 473 kHz. Additionally, it is assumed that streamlines in the upper 40% of the width of the microchannel will go to the outlet of the 2-μm microparticles, while the remaining streamlines will go to the outlet of the 2.25-μm microparticles.

**Figure 6:**
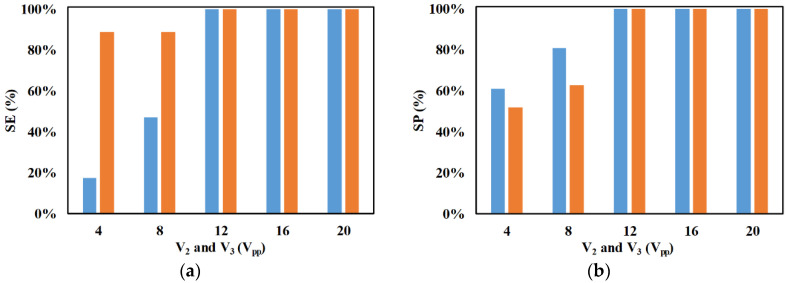
Variation of separation efficiency (SE) and separation purity (SP) with applied electrical potentials (*V*_2_ and *V*_3_) for (**a**) ■ 2 μm and (**b**) ■ 2.25 μm (*d_f_* = 60 μm, *l_f_* = 120 μm, *d_s_* = 60 μm, *l_s_* = 120 μm, *w_f_* = 6 μm, *w_s_* = 6 μm, *n_f_* = 200, *n_s_* = 200, *Q_m_* = 200 μl/h, *V*_1_ = 4 V_pp_, *V*_4_ = 4 V_pp_, *H_ch_* = 50 μm, *W_ch_* = 50 μm, 0 < *W*_*o*,2.25_ < 30 μm, 30 μm < *W*_*o*,2_ < 50 μm *N*_1_ = *N*_2_ = 10 MHz, *N*_3_ = *N*_4_ = *N*_*cr*,2_ = 473 kHz).

**Figure 7:**
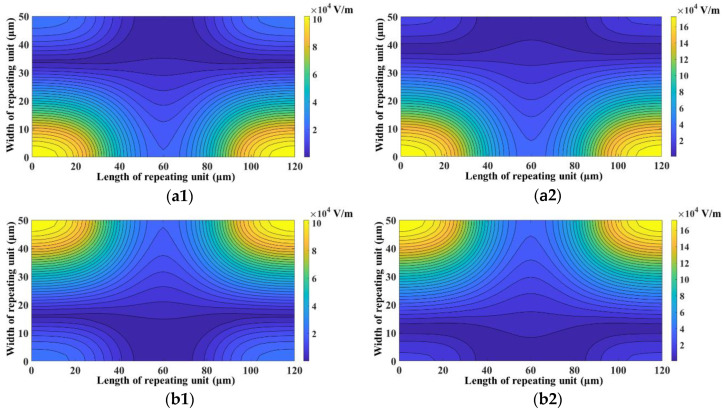
Electric field in the mid-plane along the height of (**a**) focusing section for (**a1**) *V*_2_ = 12 V_pp_ and (**a2**) *V*_2_ = 20 V_pp_ and (**b**) separation section for (**b1**) *V*_3_ = 12 V_pp_ and (**b2**) *V*_3_ = 20 V_pp_ (*d_f_* = 60 μm, *l_f_* = 120 μm, *d_s_* = 60 μm, *l_s_* = 120 μm, *w_f_* = 6 μm, *w_s_* = 6 μm, *V*_1_ = 4 V_pp_, *V*_4_ = 4 V_pp_, *H_ch_* = 50 μm, *W_ch_* = 50 μm).

Figure 6 depicts the influence of applied electrical potentials on the performance of the microfluidic device. For this study, the higher applied electrical potential of each section is varied while keeping the lower applied electrical potential constant. It is evident from Figure 6 that the increase in the differences, in the applied electrical potentials, of both sections enhances SE and SP. Figure 7 provides the schematic of the variation of electric field in the mid-plane, along the height of the microchannel, of the focusing and separation section. It can be noticed from Figure 7 that the increase in difference between the applied voltages increases the magnitude and non-uniformity of electric field, thereby leading to enhancement in the net nDEP force acting on the microparticle. The increase in the difference between the applied electrical potentials, in the focusing section, brings the 2-μm and 2.25-μm microparticles closer to the electrode pairs with lower applied electrical potential; moreover, enhancement in focusing increases the ability of the device to send the same toward its outlet. The increase in the differences between the applied electrical potential in the separation section pushes the 2.25-μm microparticles further into the region of the microchannel where streamlines move toward their outlet. It can also be noticed that, with a reduction in the difference between the applied electrical potentials, the SE of 2-μm microparticles exhibits greater deterioration than that of 2.25-μm microparticles. A reduction in the difference between applied electrical potentials reduces the degree of focusing of both 2-μm and 2.25-μm microparticles, and this reduces the number of 2-μm microparticles pushed into the region (40% of the width of the microchannel) with streamlines progressing toward the outlet of the same location. The 2.25-μm microparticles in the streamlines contained in the remaining width of the microchannel, at the end of the focusing section, as well as those 2.25-μm microparticles that are pushed into these streamlines by the nDEP force in the separation section, progress toward their outlet. Thus, the combined effects of improper focusing and the smaller contribution of the microchannel width to the outlet of 2-μm microparticles cause greater deterioration of the SE of 2-μm microparticles compared with the SE of 2.25-μm microparticles at low differences in applied electrical potentials. Consequently, the number of 2-μm microparticles reaching the outlet of 2.25-μm microparticles is greater than the number of 2.25-μm microparticles reaching the outlet of the 2-μm microparticles and, thus, the SP of 2.25-μm microparticles is lower than the SP of 2-μm microparticles at low differences between applied electrical potentials.

**Figure 8:**
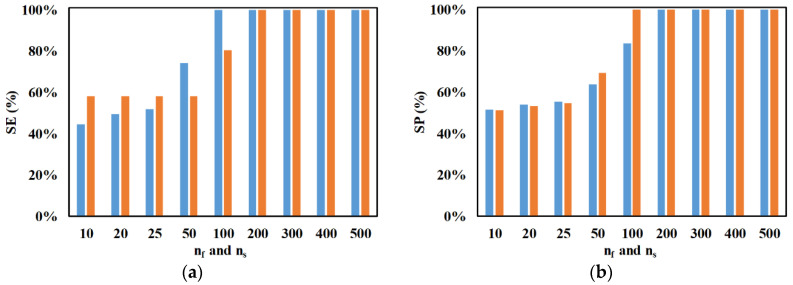
Variation of SE and SP with number of electrode pairs (n_f_ and n_s_) for (**a**) ■ 2 μm and (**b**) ■ 2.25 μm (*d_f_* = 60 μm, *l_f_* = 120 μm, *d_s_* = 60 μm, *l_s_* = 120 μm, *w_f_* = 6 μm, *w_s_* = 6 μm, *Q_m_* = 200 μL/h, *V*_1_ = 3 V_pp_, *V*_2_ = 15 V_pp_, *V*_3_ = 15 V_pp_ *V*_4_ = 3 V_pp_, *H_ch_* = 50 μm, *W_ch_* = 50 μm, 0 < *W*_*o*,2.25_ < 30 μm, 30 μm < *W*_*o*,2_ < 50 μm, *N*_1_ = *N*_2_ = 10 MHz, *N*_3_ = *N*_4_ = *N*_*cr*,2_ = 473 kHz).

Figure 8 shows the influence of the number of electrode pairs on SE and SP. It can be noticed that the increase in the number of electrode pairs increases the SE and SP of both microparticles. The increase in the number of electrode pairs increases the associated residence time, which in turn increases the duration for which the nDEP force acts on the microparticles, thereby leading to the observed enhancement in SE and SP. For a very low number of electrode pairs, the SE of 2-μm microparticles is smaller than that of 2.25-μm microparticles. At the end of the focusing section, the only 2-μm microparticles reaching their outlet are those in the streamlines of 40% of the total width of the microchannel, while 2.25-μm microparticles contained in the remaining streamlines are definitely moving toward their outlet. Another reason is the nDEP force experienced by 2.25-μm microparticles in the separation section; this causes several 2.25-μm microparticles in the streamlines progressing to the outlet of 2-μm microparticles, after the focusing section, into the streamlines progressing to the outlet of the 2.25-μm microparticles. 

**Figure 9:**
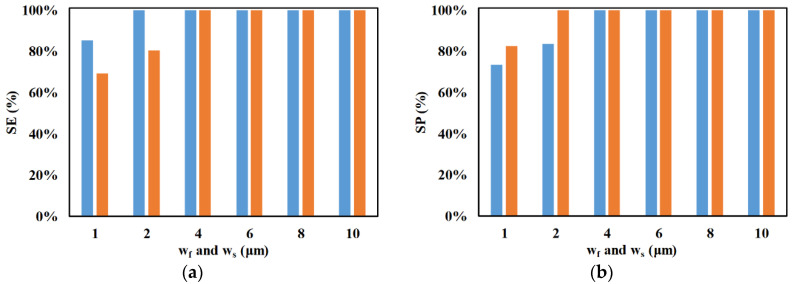
Variation of SE and SP with electrode widths (*w_f_* and *w_s_*) for (**a**) ■ 2 μm and (**b**) ■ 2.25 μm (*d_f_* = 60 μm, *l_f_* = 120 μm, *d_s_* = 60 μm, *l_s_* = 120 μm, *n_f_* = 200, *n_s_* = 200, *Q_m_* = 200 μL/h, *V*_1_ = 3 V_pp_, *V*_2_ = 15 V_pp_, *V*_3_ = 15 V_pp_ *V*_4_ = 3 V_pp_, *H_ch_* = 50 μm, *W_ch_* = 50 μm, *N*_1_ = *N*_2_ = 10 MHz, 0 < *W*_*o*,2.25_ < 30 μm, 30 μm < *W*_*o*,2_ < 50 μm*, N*_3_ = *N*_4_ = *N*_*cr*,2_ = 473 kHz).

**Figure 10:**
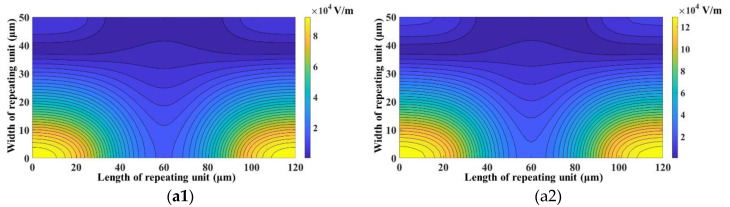

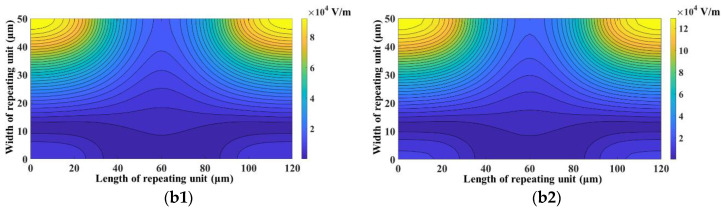
Electric field in the mid-plane along the height of (**a**) focusing section for (**a1**) *w_f_* = 2 μm and (**a2**) *w_s_* = 6 μm and (**b**) separation section for (**b1**) *w_f_* = 2 μm and (**b2**) *w_s_* = 6 μm (*d_f_* = 60 μm, *l_f_* = 120 μm, *d_s_* = 60 μm, *l_s_* = 120 μm, *V*_1_ = 3 V_pp_, *V*_2_ = 15 V_pp_, *V*_3_ = 15 V_pp_, *V*_4_ = 3 V_pp_, *H_ch_* = 50 μm, *W_ch_* = 50 μm).

Another parameter whose influence on SE and SP was analyzed is electrode width. Figure 9 shows the influence of electrode width on SE and SP. It can be clearly observed that the increase in electrode width increases SE and SP. This is because of the increase in nDEP force associated with the increase in electrode widths. The increase in electrode width increases the magnitude, as well as non-uniformity, of the electric field, which in turn increases the nDEP force. This can be clearly observed from Figure 10 which provides a comparison of the electric field inside the repeating unit of the focusing and separation sections for *w_f_* = *w_s_* = 2 μm and *w_f_* = *w_s_* = 6 μm.

**Figure 11:**
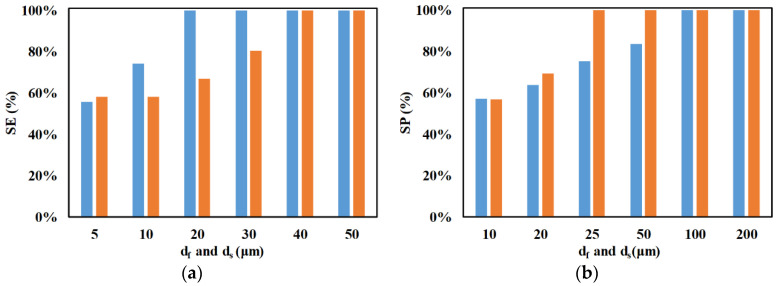
Variation of SE and SP with electrode lengths (*d_f_* and *d_s_*) for (**a**) ■ 2 μm and (**b**) ■ 2.25 μm (*w_f_* = 6 μm, *w_s_* = 6 μm, *n_f_* = 200, *n_s_* = 200, *Q_m_* = 200 μL/h, *V*_1_ = 3 V_pp_, *V*_2_ = 15 V_pp_, *V*_3_ = 15 V_pp_ *V*_4_ = 3 V_pp_, *H_ch_* = 50 μm, *W_ch_* = 50 μm, *N*_1_ = *N*_2_ = 10 MHz, 0 < *W*_*o*,2.25_ < 30 μm, 30 μm < *W*_*o*,2_ < 50 μm, *N*_3_ = *N*_4_ = *N*_*cr*,2_ = 473 kHz).

The influence of electrode lengths on SE and SP is shown in Figure 11. It can be observed that the increase in electrode lengths improves SE and SP. The increase in electrode length increases the residence time of the microparticles, as well as the magnitude of the electric potential inside the microchannel. The increase in electrode lengths increases the overall length of the device, thereby increasing the duration for which nDEP force acts on microparticles, and this is one of the reasons for the observed increase in SE and SP. Additionally, the increase in electrode length increases the magnitude of the electric potential inside the microchannel, which subsequently increases the nDEP forces experienced by the microparticles, and this is another reason for the observed increase in SE and SP.

**Figure 12:**
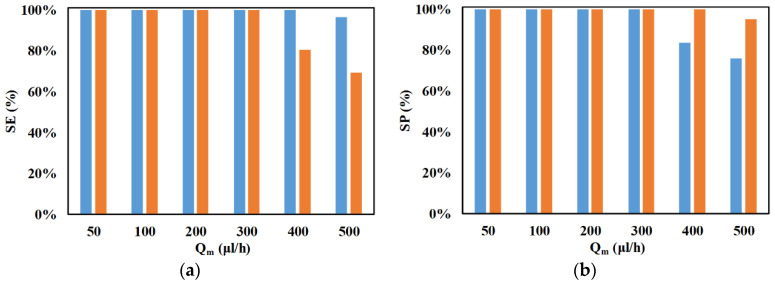
Variation of SE and SP with volumetric flowrate (*Q_m_*) for (**a**) ■ 2 μm and (**b**) ■ 2.25 μm (*d_f_* = 60 μm, *l_f_* = 120 μm, *d_s_* = 60 μm, *l_s_* = 120 μm, *w_f_* = 6 μm, *w_s_* = 6 μm, *n_f_* = 200, *n_s_* = 200, *V*_1_ = 3 V_pp_, *V*_2_ = 15 V_pp_, *V*_3_ = 15 V_pp_ *V*_4_ = 3 V_pp_, *H_ch_* = 50 μm, *W_ch_* = 50 μm, 0 < *W*_*o*,2.25_ < 30 μm, 30 μm < *W*_*o*,2_ < 50 μm, *N*_1_ = *N*_2_ = 10 MHz, *N*_3_ = *N*_4_ = *N*_*cr*,2_ = 473 kHz).

Figure 12 shows the influence of volumetric flow rate on the SE and SP of microparticles. For this study, the volumetric flow rate is varied between 50 and 500 μL/h. With an increase in volumetric flow rate, there is a reduction in the SE and SP of both microparticles. The increase in volumetric flow rate decreases the residence time of the microparticles in the microchannel, which reduces the influence of nDEP forces in positioning microparticles of both sizes, and this leads to the reduction in their SE and SP. Deterioration in the SE of 2.25-μm microparticles is observed earlier than the deterioration in the SE of 2-μm microparticles. The reduction in residence time, due to the increase in volumetric flow rate, along with the weak nDEP experienced by 2.25-μm microparticles in the separation section, is the cause of the deterioration of the SE of 2.25-μm microparticles prior to that of 2-μm microparticles. On the other hand, the SP of 2-μm microparticles deteriorates earlier than any deterioration in the SP of 2.25-μm microparticles being observed. When the SE of 2.25-μm microparticles starts to deteriorate, several 2.25-μm microparticles appear at the outlet of the 2-μm microparticles, and this is the reason for the deterioration of the SP of 2-μm microparticles initiating before that of 2.25-μm microparticles.

The efficacy of the conceptualized device is demonstrated by separating a heterogeneous mixture of 2-μm and 2.25-μm polystyrene microparticles. However, the device can be employed for separating binary heterogeneous mixtures of microparticles with sub-micron differences in size as long as the cross-over frequencies of the microparticles are different. When the cross-over frequencies are very close, the applied voltage would need to be high and a high number of electrode pairs would be required.

**Figure 13:**
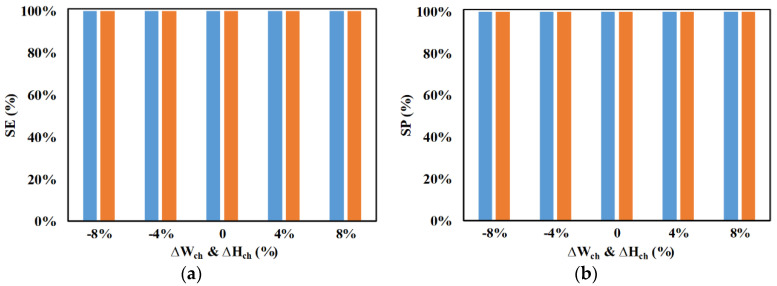
Effect of variations in width and height on SE and SP for (**a**) ■ 2 μm and (**b**) ■ 2.25 μm (*d_f_* = 60 μm, *l_f_* = 120 μm, *d_s_* = 60 μm, *l_s_* = 120 μm, *w_f_* = 6 μm, *w_s_* = 6 μm, *n_f_* = 200, *n_s_* = 200, *V*_1_ = 3 V_pp_, *V*_2_ = 15 V_pp_, *V*_3_ = 15 V_pp_ *V*_4_ = 3 V_pp_, *H_ch_* (at 0%) = 50 μm, *W_ch_* (at 0%) = 50 μm, 0 < **W*_*o*,2.25_* < 30 μm, 30 μm < *W*_*o*,2_ < 50 μm, *N*_1_ = *N*_2_ = 10 MHz, *N*_3_ = *N*_4_ = *N*_*cr*,2_ = 473 kHz).

A sensitivity study was done to understand the influence of microchannel height and width, as well as microparticle radii, on the performance metrics of the device. Figure 13 shows the influence of a simultaneous variation of width and height on the performance of the device in achieving separation. Studies were done by varying the dimensions from −8% to +8%, and the performance metrics of the same systems are compared with their performance in the absence of any variation. It can be noticed that the variation in dimensions of the microchannel does not affect the performance metrics. This behavior is very encouraging as the small variations in dimensions that are expected while creating the prototype will not affect the performance of the same system at design conditions.

**Figure 14:**
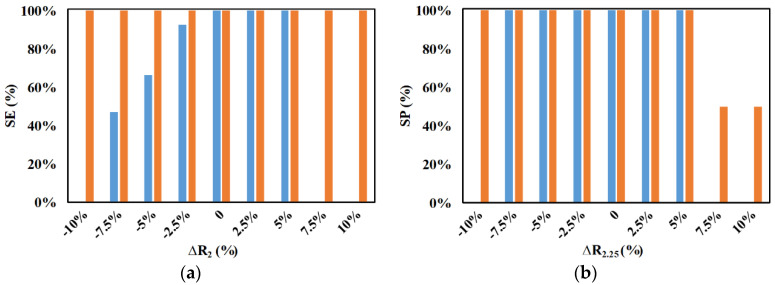
Effect of variations in radius of small microparticles on SE and SP for (**a**) ■ 2 μm (at 0%) and (**b**) ■ 2.25 μm (*d_f_* = 60 μm, *l_f_* = 120 μm, *d_s_* = 60 μm, *l_s_* = 120 μm, *w_f_* = 6 μm, *w_s_* = 6 μm, *n_f_* = 200, *n_s_* = 200, *V*_1_ = 3 V_pp_, *V*_2_ = 15 V_pp_, *V*_3_ = 15 V_pp_ *V*_4_ = 3 V_pp_, *H_ch_* = 50 μm, *W_ch_* = 50 μm, 0 < *W*_*o*,2.25_ < 30 μm, 30 μm < *W*_*o*,2_ < 50 μm, *N*_1_ = *N*_2_ = 10 MHz, *N*_3_ = *N*_4_ = *N*_*cr*,2_ = 473 kHz).

Figure 14 shows the influence of the variation of radii of the microparticles on the performance metrics of the device. Studies were done by varying the radius of the small microparticles from −10% to +10% when all other parameters are held constant, and the results are compared to those with the case of no variation in the radius of the small microparticles. It can be noticed from Figure 14a,b that the performance of the device is significantly affected when the variation in the radius of the small microparticles occurs beyond ±2.5%. When the variation is greater than −2.5%, the small microparticles start to be captured on the electrode surface, and they are prevented from the reaching their outlet. The small microparticles that are captured can be extracted by flushing the device with a buffer solution after processing the sample; however, as this is not the expected manner of operation of the device, the capturing of microparticles on the electrodes is taken to negatively affect performance metrics as observed in Figure 14a,b. When the variation in the radius of the small microparticle is as high as −10%, no microparticles appear at their exit and, thus, the SE is 0% as expected and the SP is nonexistent. When the variation in radius of the small microparticles is greater than +5%, the nDEP force they experience is high enough to push them into the streamlines moving toward the outlet of the big microparticles and, thus, the associated SE is zero and the SP is non-existent. However, as all the small microparticles appear at the outlet of the 2.25-μm microparticles, the SP of 2.25-μm microparticles for these variations is 50%.

**Figure 15:**
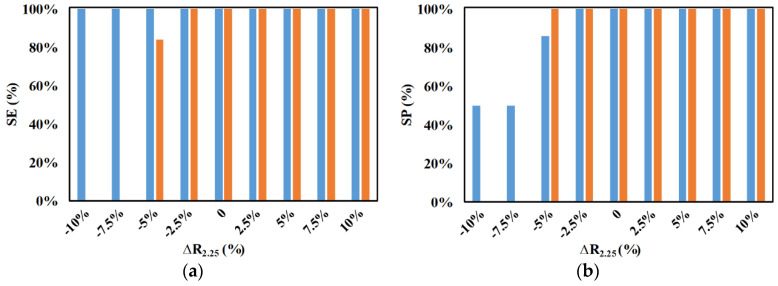
Effect of variations in radius of big microparticles on SE and SP for (**a**) ■ 2 μm and (**b**) ■ 2.25 μm (at 0%) (*d_f_* = 60 μm, *l_f_* = 120 μm, *d_s_* = 60 μm, *l_s_* = 120 μm, *w_f_* = 6 μm, *w_s_* = 6 μm, *n_f_* = 200, *n_s_* = 200, *V*_1_ = 3 V_pp_, *V*_2_ = 15 V_pp_, *V*_3_ = 15 V_pp_ *V*_4_ = 3 V_pp_, *H_ch_* = 50 μm, *W_ch_* = 50 μm, 0 < *W*_*o*,2.25_ < 30 μm, 30 μm < *W*_*o*,2_ < 50 μm, *N*_1_ = *N*_2_ = 10 MHz, *N*_3_ = *N*_4_ = *N*_*cr*,2_ = 473 kHz).

Similarly, studies were also done for the variation in radius of the big microparticles from −10% to +10% by holding all other parameters constant, and the results are compared with the case of no variation in the radius of the big microparticle as shown in Figure 15. In this case, it can be noticed that the increase in the size of the microparticles does not affect the performance metrics of the device. This is expected as the increase in size of the microparticles increases the nDEP force acting on the microparticles, thereby pushing them further in the streamlines progressing toward the outlet of the big microparticles. On the other hand, the slight reduction in the size of the big microparticles does not influence the SE and SP of the device; however, with the increase in the reduction of the radius of the big microparticles, the nDEP force experienced by the microparticles is reduced, thereby leading to them moving through the separation section unaffected and, in turn, exiting the device through the outlet of the 2-μm microparticles. This is the reason for the SE of big microparticles being zero when the variation in radius is −7.5% and −10%; the SP for these variations is non-existent. Consequently, the SP of 2-μm microparticles is 50% when the variation in radius of the big microparticles is −7.5% and −10%.

## 4. Conclusions

This article conceptualizes a dielectrophoretic microfluidic device for the separation of microparticles based on size with sub-micron resolution. The device consists of two sections; the first section termed the focusing section is dedicated to focusing of the heterogeneous sample, while the second section referred to as the separation section is dedicated to the separation of the heterogeneous sample into homogeneous samples. Both focusing and separation sections consist of two sets of independently controllable planar electrodes with each set located next to one of the sidewalls; each set of electrodes consists of multiple pairs. In the focusing section, all microparticles are focused next to one of the sidewalls, while, in the separation section, the big microparticles are pushed toward the interior of the microchannel without affecting the small microparticles, and this leads to the separation of the microparticles. A mathematical model of the conceptualized device was developed in this work. The model takes into account the several phenomena experienced by microparticles inside the device including inertia, drag, gravity, buoyancy, virtual mass, and dielectrophoresis, and it quantifies the performance of the device in terms of separation efficiency and separation purity. The model is used to demonstrate the ability of the device in achieving the separation of microparticles based on size with sub-micron resolution by separating a heterogeneous mixture of 2-μm and 2.25-μm microparticles into two homogeneous mixtures. The model was also used for a parametric study; the parameters studied include volumetric flow rate, number of electrode pairs, electrode widths, electrode lengths, and applied electrical potentials. The model is useful for designers of this particular microfluidic device, as it allows them to realize the same with desired separation efficiency and separation purity.

## Nomenclature

*d*	electrode length (μm or m)
***E***	electric field
*H*	height (m or μm)
*g*	electrode gap (μm or m)
*g_a_*	acceleration due to gravity (m/s^2^)
***F***	force vector (N)
*K_s_*	surface conductance (S)
*l*	length of repeating unit (μm or m)
*N*	operating frequency (kHz)
*m*	mass (kg)
*n*	number of electrodes (-)
*P*	pressure (Pa)
*Q*	flow rate (m^3^/s or μL/h)
*Re[f_CM_]*	Clausius–Mossotti factor (-)
*r*	radius (m or μm)
*SE*	separation efficiency
*SP*	separation purity
*t*	time (s)
***U***	velocity vector (m/s)
*u*	velocity along *x*-direction (m/s)
*V*	voltage (V)
*W*	width (m or μm)
*w*	electrode width (μm or m)
***X***	displacement vector (m or μm)
*x*	displacement in the *x*-direction (m)
*y*	displacement in the *y*-direction (m)
*z*	displacement in the *z*-direction (m)
$\bar{x}$	co-ordinate in the *x*-direction
$\bar{y}$	co-ordinate in the *y*-direction
$\bar{z}$	co-ordinate in the *z*-direction
**Greek alphabet**	
*ε*	permittivity (-)
*ε_o_*	permittivity of free space (F/m)
*σ*	conductivity (S/m)
*μ*	viscosity (Pa∙s)
*ρ*	density (kg/m^3^)
*ω*	operating frequency (rad/s)
**Subscripts**	
2	2-μm particle
2.25	2.25-μm particle
*bp*	big microparticle
*bulk*	bulk electrical conductivity
*ch*	microchannel
*cr*	cross-over
*DEP*	dielectrophoresis
*ext*	external
*e*	entity
*ext*	external
*f*	focusing section
*m*	medium
*o*	outlet
*RMS*	root mean square
*s*	separation section
*sp*	small microparticle
